# Case report: The masquerading spectrum: a pediatric case series of IgG4-related disease

**DOI:** 10.3389/fimmu.2026.1832660

**Published:** 2026-05-08

**Authors:** Olga Viktorovna Shpitonkova, Natalia Anatolievna Geppe, Vera Alekseevna Podzolkova, Elena Yurievna Afonina, Tatiana Vladimirovna Zubareva, Maria Nikolaevna Nikolaeva, Natalia Yurievna Golovanova, Maria Kirillovna Osminina, Elena Vasilievna Frolkova, Maria Alekseevna Kudryashova, Nadezhda Stepanovna Podchernyaeva

**Affiliations:** 1 Department of Children’s Diseases, Sechenov First Moscow State Medical University, Moscow, Russia; 2 Sechenov’s Center of Maternity and Childhood, Sechenov First Moscow State Medical University, Moscow, Russia

**Keywords:** children, IgG4 related disease, obliterative phlebitis, orbital pseudotumor, storiform fibrosis

## Abstract

IgG4-related disease (IgG4-RD) is a rare fibro-inflammatory condition with scarce data on its clinical spectrum and management in the pediatric population.We present a single-center observational study of six children with clinically and morphologically confirmed IgG4-RD. We analyzed the age of onset, time to diagnosis, and comprehensive clinical, imaging, laboratory, and histopathological findings at presentation and during follow-up (range: 3 months to 7 years). The 2020 revised comprehensive diagnostic criteria for IgG4-RD were used. The diagnosis was based on a biopsy of orbital tissue with detection of signs of fibrosis, obliterating phlebitis, and subsequent immunohistochemical testing for the presence of IgG4. The disease manifested itself in 4 patients with orbital pseudotumor, in 1 patient with diabetes insipidus, in 1 patient with fever, signs of high inflammatory and immunological activity. The diagnostic delay ranged from 3 to 12 months. Treatment regimens included glucocorticoids combined with cytostatic agents (n=3), cytostatic monotherapy (n=2), and initial glucocorticoid monotherapy (n=1). Three patients achieved a good clinical and radiological response without disease progression. One patient experienced multiple relapses while on topical steroid therapy, which were successfully controlled after switching to systemic cytostatic therapy. Two children achieved incomplete remission. They were advised to continue rituximab therapy. This case series underscores the diagnostic challenge and phenotypic diversity of pediatric IgG4-RD. The most common clinical manifestation in our patients was orbital involvement. Prednisone and cytostatic agents were effective for inducing and maintaining remission. Our findings contribute to the evolving understanding of this rare condition in childhood.

## 1 Introduction

IgG4-related disease (IgG4-RD) is a chronic immune-mediated fibro-inflammatory condition characterized by tumor-like tissue infiltration of IgG4-expressing plasma cells, leading to a distinctive histopathological pattern of storiform fibrosis and obliterative phlebitis (1). Since its formal nosological definition, significant progress has been made in understanding its pathogenesis, which centers on B-cell activity and a distinct T-helper-2 immune response, though the precise role of elevated IgG4 levels remains unclear (2).

Diagnosing IgG4-RD is clinically challenging due to its ability to mimic a wide spectrum of conditions, including malignancies, infections, and other autoimmune diseases. This mimicry often leads to significant diagnostic delays. The 2020 Revised Comprehensive Diagnostic (RCD) criteria, integrating clinical, serological, and pathological domains, have been established as a robust diagnostic framework (3). A critical histopathological diagnosis requires the presence of dense lymphoplasmacytic infiltrates, storiform fibrosis, and obliterative phlebitis (4).

However, the clinical landscape of IgG4-RD in the pediatric population remains poorly defined. Epidemiological data are scarce, and the disease is considered exceptionally rare in children. A literature review suggests that only approximately 20% of reported pediatric cases fully meet established adult criteria, such as the ACR/EULAR classification or the 2020 RCD criteria (5). This discrepancy highlights a substantial knowledge gap, indicating potential underdiagnosis and raising questions about whether the disease phenotype in children differs from that in adults. The heterogeneity of presentations, from organ-specific manifestations like orbital pseudotumor to systemic involvement, further complicates early recognition in this age group.

Here, we report a case series of six pediatric patients (age range 4–17 years; five females, one male) with histopathologically confirmed IgG4-RD. The series encompasses both isolated orbital disease (n=4) and systemic multi-organ involvement (n=2), illustrating the full phenotypic spectrum of this rare condition in children. We describe their clinical presentations, diagnostic workup, treatment responses, and long-term outcomes, aiming to enhance recognition and inform management of pediatric IgG4-RD.

## 2 Case description

### 2.1 Presentation and diagnosis

We present a case series of six pediatric patients (five girls, one boy) with a median age at disease onset of 12 years (range: 4–17 years). Six patients that met the following RCD 2020 criteria are summarized in **Table 1**.

**Table 1:** The 2020 RCD criteria for IgG4-RD in 6 patients.

Pt	Age/sex	Organs involved	Biopsy site	Serum IgG4, mg/dl (6, 7)	Pathology (IgG4+ cells/HPF; ratio)	Storiform fibrosis	Obliterative phlebitis	RCD 2020
1	4y, F	Left orbit, dacryoadenitis	Orbit (intraop)	22 (normal)	>25 cells; >40%	Intense	Present	Probable
2	7y, F	Bilateral orbits, lacrimal glands	Lacrimal gland (transcut)	28 (normal)	>15 cells; >40%	Moderate	Present	Probable
3	11y, F	Left lacrimal gland	Lacrimal gland (transcut)	Not performed	>20 cells; >40%	Mild	Present	Probable
4	13y, F	Left orbit, dacryoadenitis	Orbit (intraop)	17 (normal)	>10 cells; >30%	Moderate	Present	Probable
5	14y, F	Kidneys, lungs, liver, orbits, parotids	Lacrimal gland (transcut)	Not performed	>10 cells; >40%	Moderate	Present	Probable
6	17y, M	Pituitary, lungs, right orbit	Orbit (intraop)	35 (normal)	>35 cells; >40%	Moderate	Present	Probable

Pt, patient; y, years; F, female; M, male; ref, reference range; mg/dl, milligrams per deciliter; HPF, high-powered field; IgG4, immunoglobulin G subclass 4; RCD, Revised Comprehensive Diagnostic; intraop, intraoperative; transcut, transcutaneous.

According to the 2020 RCD criteria, all 6 patients met the classification of “probable” IgG4-RD. All children had organ involvement, and histopathology revealed the classic triad of lymphoplasmacytic infiltration, storiform fibrosis, and obliterative phlebitis. Serum IgG4 levels, measured after biopsy and before treatment in 4 patients, were within the normal range. In the two remaining patients, IgG4 testing was not performed: Patient 3 started therapy immediately after biopsy, and Patient 5 received immunosuppressive therapy before biopsy (Mikulicz syndrome appeared eight months later, leading to the correct diagnosis). Exclusion criteria were relevant in two patients (Patients 5 and 6), as detailed below.

The cohort’s key clinical, diagnostic, and therapeutic characteristics are summarized in **Table 2**. No clear triggering factors were identified, though two patients had a personal history of allergy, and one had a family history of systemic lupus erythematosus.

**Table 2:** IgG4-RD cohort’s key clinical, diagnostic, and therapeutic characteristics.

Pt	Age/sex	First symptoms	Diagnostic delay	Key lab/imaging	Final diagnosis (organs)	Treatment summary	Outcome
1	4y, F	Palpebral redness, swelling, ptosis	4 mo	CT: orbital mass (23x33 mm)	Orbital IgG4-RD	Pred 0.5 mg/kg (4 wk taper) + MMF 720 mg/day (6 mo) → MMF 500 mg/day (2 yr)	Remission, off therapy 4.5 yr
2	7y, F	Bilateral swelling, dacryoadenitis	6 mo	CT: bilateral orbital/lacrimal involvement	Orbital IgG4-RD	Topical steroids (8 mo, 3 relapses) → MMF 300 mg/m²/day (6 mo)	Remission on MMF
3	11y, F	Ptosis, dacryoadenitis	3 mo	CT: lacrimal mass (22x16 mm)	Orbital IgG4-RD	MMF 1000 mg/day (6 mo) → MMF 500 mg/day (1 yr)	Remission on MMF
4	13y, F	Palpebral swelling, ptosis	3 mo	CT: lacrimal mass (21x14 mm)	Orbital IgG4-RD	MMF 1000 mg/day (6 mo) → MMF 500 mg/day (1 yr)	Remission on MMF
5	14y, F	Fever, hematuria, arthralgia → later orbital/salivary swelling	8 mo	↑ESR, ALT/AST, ANA/anti-DNA; CT: lung GGO	Systemic IgG4-RD (kidneys, lungs, liver, orbits, parotids)	MP 600 mg IV ×3 → Pred 0.5 mg/kg (4 wk), then taper to 6 mg/day + CYC 600 mg/m²/mo ×3 → MMF 2000 mg/day → RTX	Partial response (Pred 6 mg/day + MMF)
6	17y, M	Polyuria/polydipsia → later orbital redness/mass	6 mo	Polyuria 4.5 L/day; ANCA+; CT: orbital mass, lung GGO	Systemic IgG4-RD (pituitary, lungs, orbit)	MP 750 mg IV ×3 → Pred 0.3 mg/kg (4 wk) + CYC 800 mg/m²/mo ×6 → RTX	Partial response (Pred 4 mg/day + RTX)

Pt, patient; y, years; F, female; M, male; mo, months; CT, computed tomography; mm, millimeters; ESR, erythrocyte sedimentation rate; ALT, alanine aminotransferase; AST, aspartate aminotransferase; ANA, antinuclear antibodies; anti-DNA, anti-double-stranded DNA antibodies; ANCA, anti-neutrophil cytoplasmic antibody; GGO, ground-glass opacities; IgG4-RD, IgG4-related disease; Pred, prednisone; MMF, mycophenolate mofetil; MP, methylprednisolone; IV, intravenous; CYC, cyclophosphamide; RTX, rituximab; mg, milligram; kg, kilogram; m², body surface area; SLE, systemic lupus erythematosus; IgE, immunoglobulin E.

**Figure 1:**
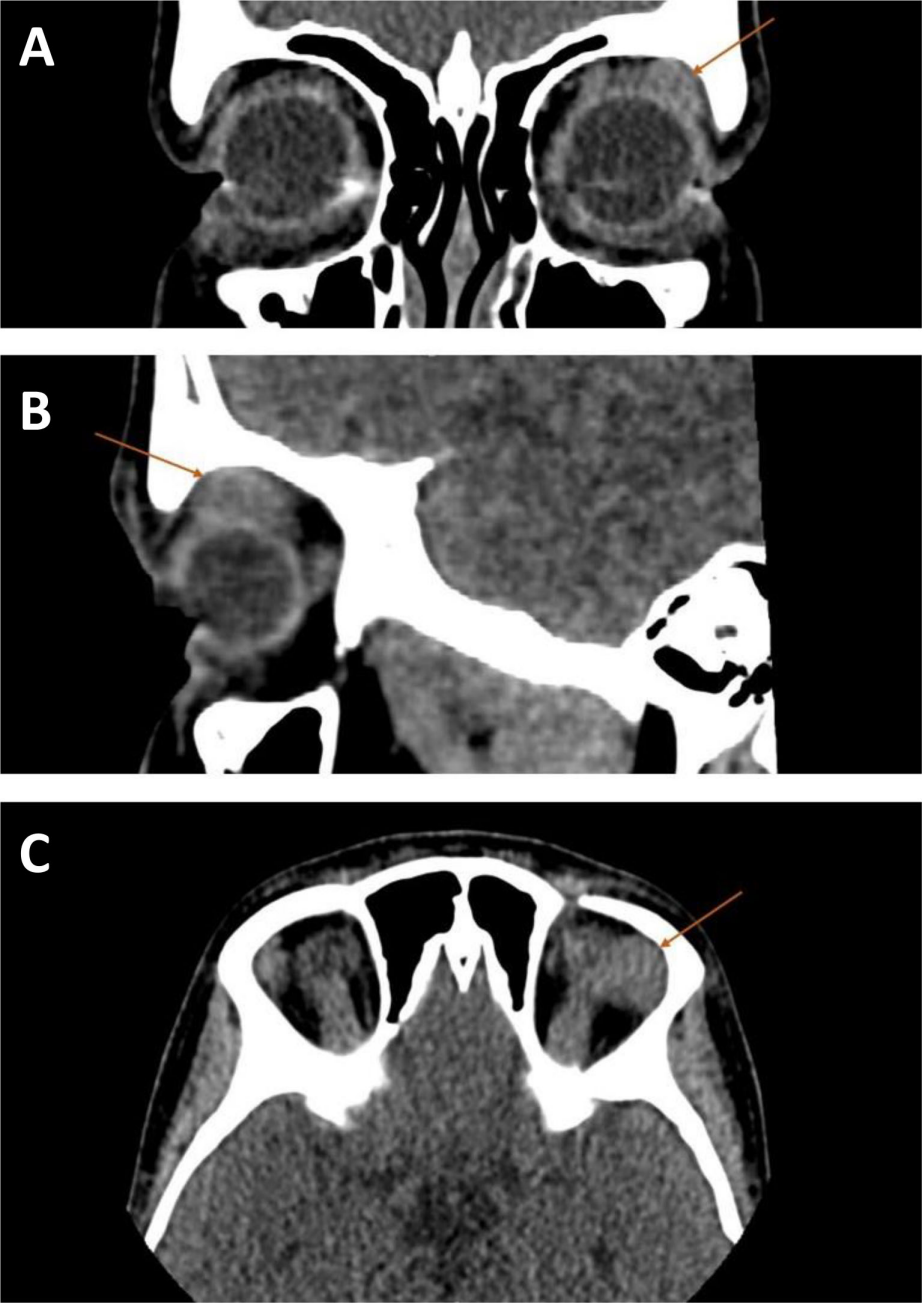
Non−contrast CT images demonstrate a soft−tissue mass in the region of the left lacrimal gland (arrows) in three orthogonal planes: **(A)** axial, **(B)** sagittal, and **(C)** coronal. The mass measures 10×13×7 mm and abuts the superolateral wall of the globe without clear invasion of adjacent soft tissues or the lateral rectus muscle. No bony destruction is observed. The imaging features were consistent with IgG4−associated ophthalmopathy, as confirmed by subsequent histopathology.

The series revealed two distinct phenotypic patterns. The first group (Patients 1-4) presented with isolated orbital disease, manifesting as eyelid swelling, redness, ptosis, and proptosis. Initial misdiagnoses included conjunctivitis, sinusitis, and orbital pseudotumor. A representative case from this group is Patient 4, a 13-year-old girl who presented with progressive left eyelid swelling and ptosis. Contrast-enhanced CT revealed a well-defined lacrimal gland mass (21.3×14 mm) with homogeneous enhancement (**Figure 1**).

Laboratory markers of inflammation and immunity were largely unremarkable in this group.

In contrast, two older patients (Patients 5 and 6) presented with complex, multi-organ systemic disease, where orbital involvement was a later finding. Patient 6 first presented with central diabetes insipidus (polyuria up to 4 L/day), initially attributed to a pituitary adenoma. Orbital inflammation appeared six months later, misleadingly suggesting two separate diseases. Patient 5 had a febrile systemic onset with arthralgia, hepatosplenomegaly, hematuria, and cough, prompting an extensive workup for vasculitis (granulomatosis with polyangiitis), lupus, and lymphoma. Orbital signs emerged eight months later. This pattern led to significant diagnostic delays of 6–12 months.

Notably, serum IgG4 levels were within normal limits in all four patients tested, including when considering age-appropriate pediatric reference ranges (6, 7), underscoring its limited diagnostic sensitivity in children. The definitive diagnosis rested on histopathology. All patients underwent a biopsy of orbital or lacrimal gland tissue: three patients with orbital lesions underwent intraoperative biopsy, the remaining three patients underwent transcutaneous orbitotomy with biopsy. All biopsy specimens were examined by immunohistochemistry (IHC). Histological examination confirmed the hallmark triad: dense lymphoplasmacytic infiltration, storiform fibrosis, and obliterative phlebitis. Immunohistochemistry revealed an IgG4+/IgG+ plasma cell ratio exceeding 40% in five patients and 30% in one.

This biopsy was crucial for re-evaluation. In Patient 6 (ANCA-positive, diabetes insipidus), biopsy specimens were reviewed twice to exclude granulomatosis with polyangiitis; the absence of granulomas ruled out vasculitis, allowing the diagnosis of IgG4-related infundibular hypophysitis. In Patient 5 (positive ANCA and anti-dsDNA antibodies), biopsy helped exclude lymphoma (negative bone marrow trephine biopsy) and other autoimmune conditions: SLE and Sjögren’s disease (negative anti-Sm, SSA/SSB, Ro/La antibodies on immunoblot), as well as autoimmune hepatitis (negative anti-LKM and anti-smooth muscle antibodies).

Post-diagnostic imaging identified subclinical visceral involvement in the two systemic cases: ground-glass opacities in the lungs (both patients) and features suggestive of IgG4-related hepatic involvement in Patient 5. Patients with isolated orbital disease had no evidence of internal organ involvement.

### Therapeutic intervention and follow-up outcomes

Treatment was tailored to disease severity (see **Table 1**). Patients with isolated orbital disease received either mycophenolate mofetil (MMF) monotherapy (n=2) or a short course of glucocorticoids bridging to MMF (n=2). Patient 2 experienced three local relapses during topical steroid therapy, which were successfully controlled after switching to systemic MMF.

The two patients with systemic disease required aggressive induction therapy. Both received intravenous methylprednisolone pulses followed by oral glucocorticoids combined with cyclophosphamide (CYC), later replaced by MMF or rituximab for maintenance.

**Figure 2:**
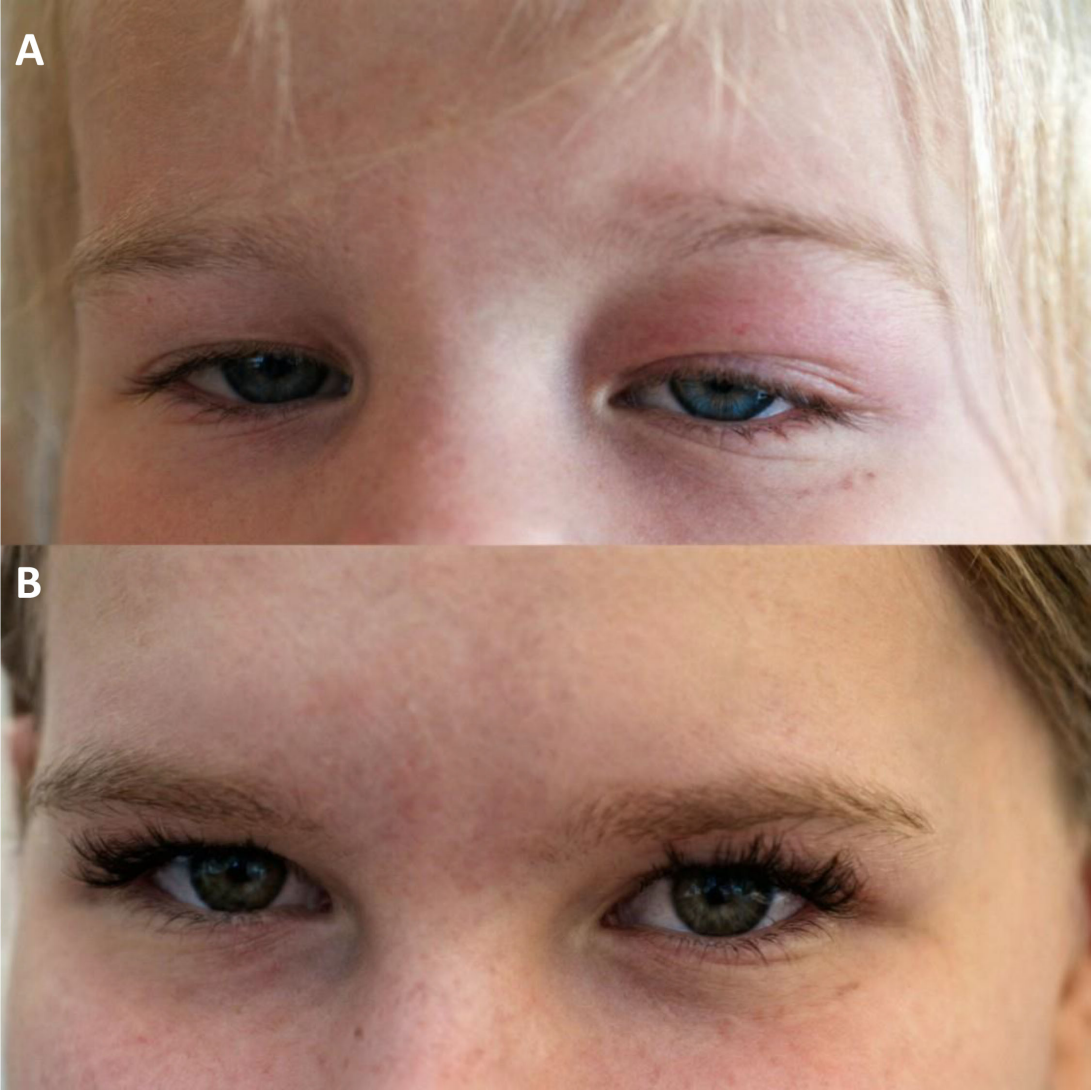
Clinical evolution of orbital involvement in Patient 1. **(A)** At diagnosis: severe upper eyelid edema, erythema, and ptosis. **(B)** After 7 years of follow-up: resolution of swelling and restoration of symmetric eye function.

At the 3-month follow-up, all patients showed a favorable response: resolution of orbital inflammation (see **Figure 2** for Patient 1) and improvement in systemic symptoms. The daily urine output in Patient 6 decreased from 4L to 2.5L. ANCA normalized, and lung opacities improved. Four patients achieved sustained drug-induced remission on maintenance MMF therapy. Two patients (the systemic cases) achieved a partial response with residual low-level serological activity and are candidates for further biologic therapy.

### Key practical points for clinicians

Based on our limited experience, we highlight the following points for suspected pediatric IgG4−RD, particularly with orbital/lacrimal involvement:

- Biopsy of affected glands or orbital mass with IHC (IgG4+/IgG+ ratio) is essential for diagnosis.
- Serum IgG4 levels are often normal in children and should not be used to exclude the disease.
- Once confirmed, imaging (CT/MRI) is recommended to screen for subclinical activity.
- Regular follow−up imaging is recommended to monitor treatment response and detect relapse.

These observations are preliminary and require validation in larger cohorts.

## 3 Discussion

This case series illustrates the diagnostic challenges and clinical heterogeneity of IgG4-RD in children, emphasizing that strict application of adult-derived serological criteria can be misleading in the pediatric population. Our findings align with the emerging literature suggesting that the phenotype and diagnostic approach to IgG4-RD in children may differ from adults (8).

Consistent with a large systematic review (64% female) (8), we observed a pronounced female predominance (5:1). The spectrum of organ involvement in our cohort supports the recognized pattern in pediatrics, where orbital disease is most frequent (4/6 patients in our series, vs. ~44% in literature) (8–10). However, our series highlights two critical complexities.

First, the systemic and masquerading presentation**s** in two older patients underscore the risk of diagnostic delay. The case of central diabetes insipidus preceding orbital disease by six months perfectly exemplifies how organ-isolated presentations can obscure the unifying diagnosis. Our experience supports the use of established criteria for “probable” IgG4-related hypophysitis in such scenarios, where biopsy of the pituitary is not feasible (11).

Second, and most notably, serum IgG4 levels were normal in all four patients tested. Published pediatric reference ranges for serum IgG4 are age-dependent and generally lower than adult norms (6, 7). Even when applying these lower pediatric thresholds, the levels in our four patients remained within the normal range, reinforcing that normogammaglobulinemia does not exclude pediatric IgG4-RD. This finding, coupled with the presence of other autoantibodies (ANCA, anti-dsDNA) in our systemic cases, directly engages with a key diagnostic controversy. While some criteria list fever and specific autoantibodies as exclusionary (3, 4), growing evidence, including recent pediatric data (12), and studies in adults (13–15), reports their co-occurrence with biopsy-proven IgG4-RD. In our patients, the absence of vasculitic granuloma on biopsy was decisive in ruling out ANCA-associated vasculitis and confirming IgG4-RD, highlighting histopathology as the ultimate.

The main strength of our case series is comprehensive histological confirmation in all six cases. Limitations include the retrospective, single-center design and small sample size. Serum IgG4 testing was not performed in two patients. Histopathology slides and original CT/MRI images were not available for publication, as investigations were performed at outside institutions. These limitations reflect the challenges of studying rare pediatric diseases and highlight the need for future multicenter studies.

Notwithstanding these limitations, our experience offers practical guidance for clinicians encountering suspected pediatric IgG4-RD. The diagnostic and therapeutic lessons from this series are fourfold. First, histopathological confirmation is indispensable, as serum IgG4 levels are often normal and thus an unreliable diagnostic screen. Second, the presence of autoantibodies such as ANCA does not exclude the diagnosis but necessitates rigorous clinicopathological review to rule out mimics. Third, treatment should be stratified: isolated orbital disease often responds to steroid-sparing agents like MMF, while systemic involvement may require more potent immunosuppression with glucocorticoids combined with cyclophosphamide or rituximab (16, 17). Finally, our series questions the sensitivity of current adult-derived diagnostic criteria in children, as most of our patients would only meet a “possible” classification. Diagnosis in pediatrics should therefore prioritize expert evaluation of classic histopathology after excluding other conditions (5, 18, 19).

## 4 Conclusion

This pediatric case series underscores that IgG4-RD is a formidable diagnostic challenge in children, often due to its masquerading clinical presentations and the limitations of standard serological tests. While orbital involvement was the most common manifestation, its delayed appearance in systemic cases and the occurrence of rare entities like infundibular hypophysitis can significantly complicate and delay the correct diagnosis. Critically, serum IgG4 levels were normal in the majority of our patients, confirming that this parameter lacks sensitivity as a standalone diagnostic criterion in the pediatric population. The definitive diagnosis invariably relied on histopathological confirmation, highlighting biopsy as the indispensable gold standard, especially in cases with atypical serology or overlapping autoantibody profiles. Although evidence-based treatment guidelines for children are lacking, our experience suggests that a tailored therapeutic approach – ranging from steroid-sparing immunosuppressants for localized disease to more aggressive regimens for systemic involvement – can effectively induce and maintain clinical remission. These observations reinforce the need for heightened clinical awareness, a low threshold for tissue biopsy, and further collaborative studies to establish pediatric-specific diagnostic and therapeutic protocols for this rare and complex immune-mediated disorder.

## Data Availability

The raw data supporting the conclusions of this article will be made available by the authors, without undue reservation.
